# Relationship among Sleep Disturbance, Stress, and Suicidal Ideation in Clinical High Risk for Psychosis

**DOI:** 10.1093/schizbullopen/sgag024

**Published:** 2026-07-08

**Authors:** Heather M Wastler, Aubrey Moe, Alexandra M Blouin, Melanie Bozzay, Melissa F V Kilicoglu, Celso Arango, Sylvain Bouix, Matthew R Broome, Monica E Calkins, Philippe Conus, Cheryl M Corcoran, Courtney Crawford, Covadonga M Diaz-Caneja, Stefano Damiani, Paolo Fusar-Poli, Matcheri S Keshavan, Vijay A Mittal, Josh Nguyen, Jason Schiffman, Jai L Shah, Walid Yassin, Alison Yung, Jean Addington, Luis Alameda, Kristin S Cadenhead, Ricardo E Carrion, Rolando I Castillo-Passi, Eric Yu Hai Chen, Jimmy Choi, Barbara A Cornblatt, Lauren M Ellman, Pablo A Gaspar, Carla Gerber, Louise Birkedal Glenthøj, Leslie E Horton, Christy Lai Ming Hui, Joseph Kambeitz, Lana Kambeitz-Ilankovic, Minah Kim, Sung-Wan Kim, Nikolaos Koutsouleris, Jun Soo Kwon, Kerstin Langbein, Daniel Mamah, Daniel H Mathalon, Merete Nordentoft, Godfrey D Pearlson, Jesus Perez, Diana O Perkins, Albert R Powers III, Jack Rogers, Fred W Sabb, Steven M Silverstein, Stefan Smesny, William S Stone, Gregory P Strauss, Judy L Thompson, Rachel Upthegrove, Swapna Verma, Jijun Wang, Daniel H Wolf, Tianhong Zhang, Ofer Pasternak, Rene S Kahn, Martha E Shenton, Patrick D McGorry, Barnaby Nelson, John M Kane, Carrie E Bearden, Scott W Woods, Accelerating Medicines Partnership® Schizophrenia (AMP® SCZ), Lauren Addamo, Uzair Ahmed, Kelly Allott, Mario Alvarez-Jimenez, G Paul Amminger, Rosie Arnold, Rebecca Beedham, Coco Beylot, Anusha Bharal, Sonia Birch, Kate Buccilli, Elle Butterworth, Eloise Cameron, John Chen, Ann Ee Ching, Beau-Luke Colton, Kate Conroy, Brittany Cook, Frederica Cooke, Simon D’Alfonso, Thomas Digby, Dominic Dwyer, Melanie Formica, Caroline Gao, Carmela Germano, Ruth Getachew, Larry Hendricks, Kevin Huynh, Sarah Joseph, Melissa J Kerr, Wing Hei Lam, Mahlee Langdon, Suzie Lavoie, Clarice Lee, Joshua Llerena, Patrick D McGorry, Brittany McQueen, Yohannes Mebrahtu, Inge Meurs, Genevieve Middleton, Neal Morrell, Adibah Amani Nasarudin, Barnaby Nelson, Elle Nguyen, Josh Nguyen, Sally O'Callaghan, Mariam Omar, Alice Partridge, Christina Phassouliotis, Matthew Phung, Andrea Polari, Marta Radosevic, Kate Reynolds, Keita Rudd, Shaheen Samnani, Isabelle Scott, Romy Schaumann, Angela Scheppokat, Raushaan Seychell, Amber Sherriff, Jashmina Shetty, Callum Snowball, Jessica Spark, Holly Still, Andrew Thompson, Michael Thwaites, Sophie Tod, Matilda Tonkovic, Cassandra M J Wannan, Thomas J Whitford, Sarah Whitson, Scott Williams, Stephen J Wood, Rochelle Ruby Ye, Sarah Youn, Hok Pan Yuen, Alison R Yung, Scott R Clark, Marlene Früh, Igor Izyurov, Juliane Korittnik, Kerstin Langbein, Véronique Mann, Constantin Siegert, Stefan Smesny, Frederick Tekook, Helle Gade Andersen, Anne Sofie Abildskov Dahl, Bjørn H Ebdrup, Nikoline Rolver Elk, Anna Feveile, Louise Birkedal Glenthøj, Kristina Ballestad Gundersen, Ulrich Lindberg, Anne Karen Nielse, Merete Nordentoft, Helle Schæbel, Mikkel Erlang Sørensen, Sofie Amalie Ege Sørensen, Daban K A Sulaiman, Matthew R Broome, Chloe Clifford, Elizabeth Farrow, Paul Hicks, Dechante Johnson, Aanya Malaviya, Aissata Mavambu, Jack Rogers, Rachel Upthegrove, Luis Alameda, Yasser Aleman, Livia Alerci, Marco Armando, Morgane Baumgartner, Martine Cleusix, Caroline Conchon, Philippe Conus, Kim Q Do, Khadimallah Ines, Raoul Jenni, Anastasie Jordan, Paul Klauser, Emylène Ostertag, Teya Petrova, DaHye Jeon, Anna Jo, Sung-Wan Kim, MiYoung Lee, ChaeWon Park, JiMin Yoo, Mei San Ang, Yi Chian Chua, Darshan Dahjalarrajah, Hui En Lisa Koh, Jue Xin Janice Koi, Bing Cai Kok, Jia Ming Lau, Jimmy Lee, Voon Hao Lew, Yu Juan Lim, Siwei Cisy Liu, Wen Liang Loh, Amirah M Iskandar, Kwun Kei Eric Ng, Gurpreet Rekhi, Nathanael Tang, Yu Zheng Charmaine Tang, Yinghui Teng, Sin Yi Joice Tham, Pushpalata Tharumalingam, Swapna K Verma, Jie Yin Yee, Hui Hui Michelle Yeo, Yichi Zhang, Juan Helen Zhou, Candice Yu Hay Chan, Eric Yu Hai Chen, Lai Ming Christy Hui, Sonia Suet Ching Lo, Hui Lam Ng, Melody Miriam So, Yi Nam Suen, Ming Fung Tang, Tsz Lam Miriam Yeung, Öznur Bastürk, Annkathrin Böke, Millennia Chakraborty, Sophie Fengler, Lana Kambeitz-Ilankovic, Joseph Kambeitz, Sophie Müller, Julian Wenzel, Tatiana Adasme, Alejandro Bruna, Rolando I Castillo-Passi, Pamela Contreras, Sebastián Corral, Pablo A Gaspar Ramos, Alejandro Maturana-Hurtado, Rocío Mayol-Troncoso, Pamela Paz, Manuel Reyes, Cesar Salinas, Gabriel Turra-Fariña, Daniela Valle, Francisco Zamorano, David Cotter, David Mongan, Jessica Hartmann, Naomi Wray, Hanneke Wigman, Laura L Adery, Carrie E Bearden, Monica E Done, Gil D Hoftman, Dylan E Hughes, Kristen Laulette, Bernalyn Ruiz-Yu, Jamie L Zinberg, Rui Ma, Jennifer Betancourt, Vardui Grigoryan, Sparsh Moondhra, Vanessa Calderon, Simon Kapler, Katherine Karlsgodt, Andrea Vazquez Espinosa, Daisy Lopez, Oliver Flores, Misty C Richards, Debha Amatya, Danielle Denenny, Seohyun Joo, Sheena Salonga, Emily Martinez, Rachel McKinney, Jared Gilbert, Sergio Duron, Maria Golchin, Lorena Ruvalcaba, Grace Cai, Zachary Wong, Neda Awad, Daisy Lopez, Tanya Diaz, Mary Rshtouni, Meghan Kussman, Monica Espana-Velasquez, Sullivan Salone, Wendy Zhang, Victoria Rosen, Lauren Trinidad, Alexis Worthley, Angela Jin, Remy Noveshen, Lisa Bartolomeo, Juliet Hagar, Haley Wang, Katherine Byrne, Rhideeta Jalal, Jee Won Kang, Masha Kotlyarevskiy, Dinisha Mingo, Arianna Mordy, Lingyan Yu, Brittney Sy, Petra Rupert, Melodie Yen, Brittany Wolff, Ella Bogomilsky, Carla Gerber, Fred W Sabb, Carli Smythe, Nicole Swann, Meghan Davis-Reyes, Brett Barnes, Kelsey Schultz, Alexander Rockhill, Caghain McCoy, Daniel Brown, Jolinda Smith, Apoorva Karekal, Vince Quesada, Alex Holmes, Coleen Hudkins, Nicolas R Bolo, Andrew (Jin Soo) Byun, Matcheri Keshavan, Erlend Lane, Jane Mikkelson, Carsten Langholm, Setari Parsa, Gennarina Santorelli, William S Stone, John Torous, Michelle Friedman-Yakoobian, Camille Evans, Walid Yassine, Highsmith Rich, Nikita Bansal, Margret Niznikiewicz, Lynda Tucker, Colette Potts, Danielle Larsen, Debra Tolstonog, Kalina Sabeva, Courtney Crawford, Siyan He, Siyu Li, Olivia Aveson, Raquelle Mesholam-Gately, Jean Addington, Amy Shalev, Araba Chintoh, Bradley Goodyear, Cari Jahraus, Emma Truscott, Erin Jones, Lu Liu, Karl Jungert, Monica Chu, Paul Romo, Amanda Chao, Dominique Bonneville, Jacqueline Stowkowy, Kali Brummitt, Kristin Minara, Ruth Olsen, Timothy Michaels, Xiang He, Michael Birnbaum, Mitchell Arnovitz, Andrea Auther, Ricardo E Carrión, Barbara A Cornblatt, John M Kane, Patricia J Marcy, Priya Matneja, Danielle McLaughlin, Susan Ray, Lauren Serrano, Nilda Rivera-Reyes, Rachel F Bleggi, Clark D Jeffries, Diana O Perkins, Jenna Barbee, Sree Gogineni, Abigail Shuman, Amanda Neal, Cecilia Pearson Sousa, Daphney Bryan, Earl McBride, Elena Pokowitz, Rohan Kumar, Amber Leinward, Aysenil Belger, Brittany Weir, Calli Holshouser, Camila Vallebona, Christian Santos, Daniel Kutuzov, Emily Hammer, Idil Baran, Jennifer Nieri, Karen Ecsedy, Nicholas Sheppard, Rachel Bleggi, Shreeya Yarlagadda, Soliha Norbekova, Victor Pincay, Erica Oake, Averi Goldman, Emilie Powell, Marissa Mulea, Asher Alexander, Ewa Zapala, Jasper Mark, Sevde Aksoy, Erika Allison, Claire Deen, Anjali Chandrasekhar, Aidan Blumsack, Eliza List, Catherine Spencer, Danielle Pratt, Franchesca Kuhney, Gabrielle Olson, Isabelle Frosch, Ivanka Ristanovic, Jessica Fattal, Joanna Hernandez, Julie Kwon, Katherine Damme, Luz Maria Alliende Serra, Madison Stevens, Sophia Parmacek, Stew Shankman, Trevor Williams, Vanessa Zarubin, Vijay Mittal, Trevor Williams, Cameron Martinez, Chloe Krugel, Erica Karp, Gillian Ho, Jadyn Park, Juston Osborne, Kathy Moran, Kelly Bates, Maksim Giljen, Zachary Anderson, Kristin S Cadenhead, Jiayi Lin, Vereena Metry, Leda Kennedy, Heline Mirzakhanian, Cherine Akkari, Tyrone D Cannon, Youngsun T Cho, Nicole Santamauro, Kangjoo Lee, Masih Rahmati, Jie Lisa Ji, Catalina V Mourgues, Angela R Nunez, Munaza Ali, Alan Anticevic, Clara Fonteneau, Karlos J Mate Piovanetti, Holly Enden, Jenna Gannon, Brandon Asika Etuka, Godfrey D Pearlson, Albert R I I I Powers, Vinod H Srihari, Zailyn Tamayo, Emily Farina, Mara Heneks, Samuel Brege, Barbara C Walsh, Scott W Woods, Alvaro Granados, Seyenah Lopez, Terry Camarro, John Cahill, Holly K Hamilton, Daniel H Mathalon, Spero C Nicholas, Lenie Torregrossa, Renée Dembo, Zoe Rygg, Annalise Jear, Stephanie Haft, Emily Parsons, Cindy Herrera, Marvin Ruan, Cassandra Marzke, Janet Hwang, Sarah Eteminan, Frederikke Elnegaard, Chi C Lao, Paul A Betancourt, Akemi Mii, Chris Perry, Brian Roach, Stephanie Stiavetti, Barbara Stuart, Alexandra Barnes, Alyssa Bathery, Monica E Calkins, Raquel E Gur, Ruben C Gur, Irena Kesselring, Christian G Kohler, David R Roalf, Kosha Ruparel, Sage Rush, Chloe Savage, Bruce I Turetsky, Daniel H Wolf, Eirini Zoupou, Julia Milkins, Lily Savage, Natalia Sonsin-Diaz, Eli Kales, Naomi Shifman, Nick Wellman, Nicole Kim, Allison Port, Alexandria Selloni, Dewey Chu, Gaurav Verma, Margaret Garret, Muhammad Parvaz, Riaz Shaik, Shaynna Herrera, Yulia Landa, Daniel Sauermilch, Byron Ramirez, Violet (Jingyi) Yang, Tahlya Holness, Elizabeth Ray, Pojen Deng, Meghana Gogineni, Qiwen Gu, Abigayle Fogarty, Katherine Dokhoylan, Elana Gabriel, Ronit Shvarzman, Agrima Srivastava, Angela Lu, Antigone Phili, Cansu Sarac, Conrad Musey, Ezra Ellenberg, Jane Gorman, Julia Norman, Matthew Cotter, Maxwell Mikelic, Nahal Talasazan, Sophia Shuster, Zachary Bergson, Nicole Hays, Lea Erjavc, Cheryl M Corcoran, Rene S Kahn, Alessia McGowan, Abraham Reichenberg, Agrima Srivastava, Inge Winter-van Rossum, Tina Gupta, Leslie E Horton, Rebecca McIlhenny, Christian Porter, Charise Peters, Dean Salisbury, Emma Headley, Fabio Ferrarelli, Hannah Kirsch, Maria Jalbrikowski, Megan Deam, Thomas, AnaKarina Villa, Salsabil Gehan, Syndey Barlow, Sara McNemar, Lauren M Ellman, Kyle S Kinney, Amelia McDonnell, Joshua E Mervis, Vishnu Murty, Raana Mohyee, Stephanie Korenic, Ian Ogborn, Brennan Burns, Emily Lipner, Rachel Furlan, Anjani Ningaiah, Blake Elliot, Bry Estupinan, Ellen Whitton, Emma Smith, Jacqueline Wurman, Jay Hope, Katey O'Brien, Lily Towle, Madeline Pike, Nina Bertolami, Olivia Garces, Riley Capizzi, Shelby Tkacik, Thomas Olino, Vishnu Murty, Zeeshan Huque, Ranesh Mopuru, Raayana Arora, Johanna M Jarcho, Zarina R Bilgrami, Tae'lar Henry, Linying Li, Elaine Walker, Phillip Wolff, Gregory P Strauss, Ashley Zollicoffer, Delaney Collins, Gifty Ayawvi, Lauren Arnold, Lauren Jennings, Lisa Bartolomeo, Luyu Zhang, Melissa McDowell, Sierraann Jarvis, Stephanie Croyle, Sydney James, T J Hardy, Zachary Carter, Zhixin Zhang, Masoomeh Faghankhani, Michael P Harms, Daniel Mamah, Aakash Patel, Andrey Anokhin, Jacob Woessner, Joshua Shimony, Leonard Imbula, Malana Kanallakan, Megan Sneed, Semyon Golosheykin, ShingShiun Chen, Ginger Nicol, Alaina Yarber, Ashley Meyer, Janine Bijsterbosch, Jerome Nashed, Miranda Smith, Ozlem Korucuoglu, Rebekka Hakestad, Reza Hamidian, Shalaka Nimmagadda, Shayna Rosen, Yazen Al-Hosni, Christina Pritchard, Lisa Callison, Ruben Ybarra, Adrian Preda, Alison Boos, Brandi Rollins, Elizabeth Martin, Emily Petti, Jason Schiffman, Karen Coronado, Madeline Snyder, Maksim Giljen, Mia Villegas, Miranda Bridgwater, Theo van Erp, Yerim Ryu, Lindsay Healey, Monica Esquer, Hannah Moring, Marco Maldonado, Michelle Zernick, Arlene Dominguez, Ceouna Hegwood, Angelo Richardson, Deirdre von Pechmann, Diana King, Godfrey D Pearlson, Jadwiga Collen, Jennifer Scagliotti, Jennifer Zajac, Jesse Charpentier, Michael Stevens, Patricia Graham, Russell Starankewicz, Sara Putala, Isabelle Genereux, Elizabeth Hendry, Caitlyn Begley, Jimmy Choi, Aarti Kotecha, Laci-Ann Mosher, Frescia Velarde, Samantha Aversa, M Mallar Chakravarty, Martin Lepage, Rachel A Rabin, Jai L Shah, Elissa Zavaglia, Brianna Beesley, Fjolla Berisha, Joelle Eid, Xing Dai, Felicia Proteau-Fortin, Joseph Ghanem, Diana Berjaoui, Gareth Barker, Matthew Kempton, Candida Vasconcelos, Andrea DeMicheli, Tom Spencer, Amedeo Minichino, Sebastien Brodeur, Ilaria Toniol, Andrés Estradé, Paolo Fusar-Poli, Sarah Kerins, Renato Borgatti, Stefano Damiani, Martina Maria Mensi, Anna Pichiecchio, Pierluigi Politi, Umberto Provenzani, Ilaria Bonoldi, Paolo Fusar-Poli, Celso Arango, Covadonga M Diaz-Caneja, Alvaro Andreu-Bernabeu, Adrian Capllonch, Ana Sanchez-Camara, Santiago Gil-Molina, Isabel Morales, Celia Ordas, Hayford Abrokwa, Joaquin Galvan, Julio Prieto-Montalvo, Jose Suárez-Campayo, Maria Lucas, Marta Sepúlveda-Palomo, Jessica Merchán-Naranjo, Pablo Andres-Camazon, Raquel Rodriguez, Juan Alvarez-Linera, Vito Cavone, Sandra Recio, Ana Echevarria, Violeta Casas, Rebeca Toledano, Miriam Ayora, Renzo Abregu-Crespo, Teresa Bollain, Liliana Galindo, Jesus Perez, Harri Allan, Izabele Batkovskyte, James Hughes, Julia Szacilo, Laura Stolp, Nicola Marshall, Tristan Bekinschtein, Vicky Lupson, Lucy Slade, Ben Cook, Nikolaos Koutsouleris, Alexandra Tchinitchian, Anne Ruef, Beate Dornheim, Caroline Plett, Christopher Eberle, Clara Weyer, Daniel Keeser, John Fanning, Lisa-Maria Neuner, Nadia Bieler, Nicole Obermuller, Oliver Pogarell, Peter Zill, Ronja Stohmann, Sabine Sirch, Sylvia de Jonge, Maria Urquijo, Elif Sarisik, Nestor Germanos, Jun Soo Kwon, Eugenie Choe, Harin Oh, Inkyung Park, Minah Kim, MinJi Ha, Sang Jee Rhee, Sunah Choi, Ji Seon Jang, Nayoung Sung, Heera Kim, Sunghyun Park, Jun Seo Hwang, Moonyoung Jang, Sori Kim, HyunGyou Park, Jong Rak Kim, Jungha Lee, Jiwon Son, Yeonjin Lee, Cho Won Ham, DongJin Shin, JongWhan Kang, Sulim Jeong, Sumin Yeo, SuYong Lee, TakWan Kim, Won Lee, Woori Choi, WuJeong Hwang, YooBin Kwak, YuRim Shim, SuJin An, Nicholas J K Breitborde, Ivy F Tso, Jessica A Turner, Heather M Wastler, Nancy Lundin, Sarah Hamilton, Cassandra Hennes, Vindhya Srikanth, Nichole Storey, Raffles (Henry) Cowan, Catherine Baughman, Alexandra Blouin, Aubrey Moe, Craig Parris, Sydney Craig, Callie Orme, Walter Stearns, Hossam Guigis, Amanda Kostayak, Judy L Thompson, Steven M Silverstein, Brittany Blose, Clifton Miller, Iwona Juskiewicz, Lyvia Bertolace, Madalina Tivarus, Pamela Zachary, Susan Wall, Tsion Eshetu, Dominic Oliver, Ameneh Asgari-Targhi, Adrianna Belskis, Tashrif Billah, Owen Borders, Lisa Brown, Evan Gentis, Dylan Campbell, Holly Carrington, Suheyla Cetin-Karayumak, Kelly Hay Yee Chan, Justine Chen, Kota Chin, Kang Ik K Cho, Michael J Coleman, Shreyas Fadnavis, Anastasia Haidar, Grace R Jacobs, Omar John, Tina Kapur, Sinead Kelly, Nicholas Kim, Elana Kotler, Marek Kubicki, Maria Loy, Dheshan Mohandass, Lipeng Ning, Ofer Pasternak, Nora Penzel, Nicholas Prunier, Yogesh Rathi, Hannah Reiter, Caitlin Ridgewell, Johanna Seitz-Holland, Martha E Shenton, Mark Vangel, Simone Veale, Alana Wickham, Justin T Baker, Yoonho Chung, Habiballah Rahimi Eichi, Michaela Ennis, Kathryn E Lewandowski, Einat Liebenthal, Eric Lin, Beier Yao, Cheryl M Corcoran, Rene S Kahn, Alessia McGowan, Abraham Reichenberg, Agrima Srivastava, Inge Winter-van Rossum, Sylvain Bouix, Robert J Glynn, Alicia Martin, Felecia Cerrato, Adrianna Maglieri, Lerato Majara, Mathias Goncalves, Christopher Markiewicz, Russell A Poldrack, Gregory A Light, Daniel H Mathalon, Spero C Nicholas, Carla Agurto, Eduardo Castro, Guillermo Cecchi, Pablo Polosecki, Jenna M Reinen, Qingqin S Li, Gahan J Pandina, Srinivasan Vairavan, Seth Hopkins, Sharin Roth, Michael S Sand, Terri Brister, Ken Duckworth, Carlos A Larrauri, Christoph von der Goltz, Glen Wunderlich, Sara J Shnider, Brandon Staglin, Sophia Frangou, Arundati Nagendra, Lynsey Bilsland, Loni Ajagbe, Sri Ramulu Pullagura, Martin Sabandal, Alessio Travaglia, Patricia Arean, Shelli Avenevoli, Linda Brady, Gregory Farber, Suzanne Garcia, Robert Heinssen, Sarah (Holly) Lisanby, Sarah Morris, Laura Rowland, Ming Zhan, Tiffany Farchione, Bernard Fischer, Valentina Mantua, Nicholas Breitborde

**Affiliations:** Department of Psychiatry and Behavioral Health, The Ohio State University, Columbus, OH, 43210; Early Psychosis Intervention Center (EPICENTER), The Ohio State University, Columbus, OH, 43210; Department of Psychiatry and Behavioral Health, The Ohio State University, Columbus, OH, 43210; Early Psychosis Intervention Center (EPICENTER), The Ohio State University, Columbus, OH, 43210; Department of Psychiatry and Behavioral Health, The Ohio State University, Columbus, OH, 43210; Early Psychosis Intervention Center (EPICENTER), The Ohio State University, Columbus, OH, 43210; Department of Psychiatry and Behavioral Health, The Ohio State University, Columbus, OH, 43210; Department of Psychiatry and Human Behavior, Brown University, Providence, RI, 02912; Department of Psychiatry and Behavioral Health, The Ohio State University, Columbus, OH, 43210; Early Psychosis Intervention Center (EPICENTER), The Ohio State University, Columbus, OH, 43210; Department of Child and Adolescent Psychiatry, Institute of Psychiatry and Mental Health, Hospital General Universitario Gregorio Marañón, IiSGM, CIBERSAM, Instituto de Salud Carlos III, School of Medicine, Universidad Complutense, 28009, Madrid, Spain; Department of Software Engineering and Information Technology, École de technologie supérieure, Montréal, H3C 1K3, QC, Canada; Department of Psychiatry, Brigham and Women's Hospital and Harvard Medical School, Boston, MA, 02115, United States; Institute for Mental Health, University of Birmingham, B152TT, Birmingham, United Kingdom; Early Intervention for Psychosis Service, Birmingham Women’s and Children’s NHS Foundation Trust, B138QE, Birmingham, United Kingdom; Department of Psychiatry, Perelman School of Medicine, University of Pennsylvania, Philadelphia, PA, 19143, United States; Service de Psychiatrie Générale Dép, de Psychiatrie CHUV Lausanne, 1003, Lausanne, Switzerland; Department of Psychiatry, Icahn School of Medicine at Mount Sinai, New York, NY, 10029, United States; Department of Psychiatry, Beth Israel Deaconess Medical Center, Harvard Medical School, Boston, MA, 02215, United States; Department of Child and Adolescent Psychiatry, Institute of Psychiatry and Mental Health, Hospital General Universitario Gregorio Marañón, IiSGM, CIBERSAM, Instituto de Salud Carlos III, School of Medicine, Universidad Complutense, 28009, Madrid, Spain; Department of Brain and Behavioral Sciences, University of Pavia, 27100, Pavia, Italy; Department of Brain and Behavioral Sciences, University of Pavia, 27100, Pavia, Italy; Department of Psychosis Studies, King's College, SE58AF, London, United Kingdom; Department of Psychiatry, Beth Israel Deaconess Medical Center, Harvard Medical School, Boston, MA, 02215, United States; Department of Psychology, Northwestern University, Evanston, IL 60208, United States; Centre for Youth Mental Health, The University of Melbourne, Parkville, VIC, Australia; Orygen, 3052, Parkville, VIC, Australia; Department of Psychological Science, University of California, Irvine, CA, 92697, United States; PEPP-Montreal, Douglas Research Centre, H4H 1A8, Montreal, QC, Canada; Department of Psychiatry, McGill University, H4H 1R3, Montreal, QC, Canada; Department of Psychiatry, Beth Israel Deaconess Medical Center, Harvard Medical School, Boston, MA, 02215, United States; Institute of Mental and Physical Health and Clinical Translation (IMPACT), Deakin University, 3220, Geelong, VIC, Australia; School of Health Sciences, University of Manchester, M14 4PX, Manchester, United Kingdom; Department of Psychiatry, Hotchkiss Brain Institute, University of Calgary, Calgary, AB, T2N 4Z6, Canada; Service de Psychiatrie Générale Dép, de Psychiatrie CHUV Lausanne, 1003, Lausanne, Switzerland; Department of Psychosis Studies, King's College, SE58AF, London, United Kingdom; University of California, San Diego, CA, 92093, United States; Department of Psychiatry, Donald and Barbara Zucker School of Medicine, Hempstead, NY, 11549, United States; Institute of Behavioral Science, Feinstein Institutes for Medical Research, Northwell Health, Manhasset, NY, 11030, United States; Department of Psychiatry, IMHAY, University of Chile, 8330111, Santiago, Chile; Department of Neurology and Psychiatry, Clínica Alemana - Universidad del Desarrollo, 7610658, Santiago, Chile; Department of Psychiatry, University of Hong Kong, Hong Kong, China; Olin Neuropsychiatry Research Center, Hartford HealthCare Behavioral Health Network, Hartford, CT, 06106, United States; Department of Psychiatry, Donald and Barbara Zucker School of Medicine, Hempstead, NY, 11549, United States; Institute of Behavioral Science, Feinstein Institutes for Medical Research, Northwell Health, Manhasset, NY, 11030, United States; Department of Psychology & Neuroscience, Temple University, Philadelphia, PA, 19122-6085, United States; Department of Psychiatry, IMHAY, University of Chile, 8330111, Santiago, Chile; Prevention Science Institute, University of Oregon, Eugene, OR, 97401, United States; Copenhagen Research Centre for Mental Health, Mental Health Copenhagen, Department of Psychology, University of Copenhagen, 2900, Copenhagen, Denmark; Department of Psychiatry, University of Pittsburgh School of Medicine, Pittsburgh, PA, 15213, United States; Department of Psychiatry, University of Hong Kong, Hong Kong, China; University of Cologne, Faculty of Medicine and University Hospital of Cologne, 50937, Cologne, Germany; University of Cologne, Faculty of Medicine and University Hospital of Cologne, 50937, Cologne, Germany; Department of Neuropsychiatry, Seoul National University Hospital, Seoul, 03082, South Korea; Department of Psychiatry, Seoul National University College of Medicine, Seoul, 03080, South Korea; Department of Psychiatry, Chonnam National University Medical School, Gwangju, 61469, South Korea; Mindlink, Gwangju Bukgu Mental Health Center, Gwangju, South Korea; Department of Psychosis Studies, King's College, SE58AF, London, United Kingdom; Department of Psychiatry and Psychotherapy, Ludwig-Maximilian-University Munich, 80802, Munich, Germany; Department of Psychiatry, Seoul National University College of Medicine, Seoul, 03080, South Korea; Department of Psychiatry and Psychotherapy, Jena University Hospital, 07743, Jena, Germany; Department of Psychiatry, Washington University School of Medicine, St. Louis, MO, 63110, United States; Department of Psychiatry and Behavioral Sciences and Weill Institute for Neurosciences, University of California, San Francisco, San Francisco, CA, 94158, United States; Mental Health Service 116D, Veterans Affairs San Francisco Health Care System, San Francisco, CA, 94121, United States; Mental Health Services in the Capital Region, 2900, Copenhagen, Denmark; Department of Clinical Medicine, Copenhagen University Hospital, 1353, Copenhagen, Denmark; Olin Neuropsychiatry Research Center, Hartford HealthCare Behavioral Health Network, Hartford, CT, 06106, United States; Department of Psychiatry, Yale University School of Medicine, New Haven, CT, 06519, United States; CAMEO, Early Intervention in Psychosis Service, Cambridgeshire and Peterborough NHS Foundation Trust, SE58AZ, Cambridge, United Kingdom; Department of Medicine, Institute of Biomedical Research (IBSAL), Department of Medicine, Universidad de Salamanca, 37007, Salamanca, Spain; Department of Psychiatry, University of North Carolina at Chapel Hill, Chapel Hill, NC, 27514, United States; Department of Psychiatry, Yale University School of Medicine, New Haven, CT, 06519, United States; Connecticut Mental Health Center, New Haven, CT, 06519, United States; Institute for Mental Health, University of Birmingham, B152TT, Birmingham, United Kingdom; Centre for Human Brain Health, University of Birmingham, B15 2TT, Birmingham, United Kingdom; Department of Psychiatry, IMHAY, University of Chile, 8330111, Santiago, Chile; Department of Psychiatry, University of Rochester Medical Center, Rochester, NY, 14642, United States; Department of Psychiatry and Psychotherapy, Jena University Hospital, 07743, Jena, Germany; Department of Psychiatry, Beth Israel Deaconess Medical Center, Harvard Medical School, Boston, MA, 02215, United States; Department of Psychology, University of Georgia, Athens, GA, 30602-3013, United States; Department of Psychiatry, University of Rochester Medical Center, Rochester, NY, 14642, United States; Early Intervention for Psychosis Service, Birmingham Women’s and Children’s NHS Foundation Trust, B138QE, Birmingham, United Kingdom; Department of Psychiatry, Medical Sciences Division, University of Oxford, OX3 7JX, Oxford, United Kingdom; Institute of Mental Health, 539747, Singapore, Singapore; Shanghai Mental Health Center, Shanghai Jiaotong University School of Medicine, Shanghai, China; Department of Psychiatry, Perelman School of Medicine, University of Pennsylvania, Philadelphia, PA, 19143, United States; Shanghai Mental Health Center, Shanghai Jiaotong University School of Medicine, Shanghai, China; Department of Psychiatry, Brigham and Women's Hospital and Harvard Medical School, Boston, MA, 02115, United States; Department of Radiology, Brigham and Women's Hospital and Harvard Medical School, Boston, MA, 02115, United States; Department of Psychiatry, Icahn School of Medicine at Mount Sinai, New York, NY, 10029, United States; Department of Psychiatry, Brigham and Women's Hospital and Harvard Medical School, Boston, MA, 02115, United States; Centre for Youth Mental Health, The University of Melbourne, Parkville, VIC, Australia; Orygen, 3052, Parkville, VIC, Australia; Centre for Youth Mental Health, The University of Melbourne, Parkville, VIC, Australia; Orygen, 3052, Parkville, VIC, Australia; Department of Psychiatry, Donald and Barbara Zucker School of Medicine, Hempstead, NY, 11549, United States; Institute of Behavioral Science, Feinstein Institutes for Medical Research, Northwell Health, Manhasset, NY, 11030, United States; Department of Psychiatry and Biobehavioral Sciences & Psychology, Semel Institute for Neuroscience and Human Behavior, University of California, Los Angeles, Los Angeles, CA, 90095, United States; Department of Psychiatry, Yale University School of Medicine, New Haven, CT, 06519, United States; Connecticut Mental Health Center, New Haven, CT, 06519, United States; Department of Psychiatry and Behavioral Health, The Ohio State University, Columbus, OH, 43210; Early Psychosis Intervention Center (EPICENTER), The Ohio State University, Columbus, OH, 43210

**Keywords:** suicide risk, clinical high risk, psychosis, sleep, stress

## Abstract

**Background and Hypothesis:**

Sleep disturbance is a well-established risk factor for suicide, though few studies to date have examined whether sleep disturbance contributes to suicide risk among individuals at clinical high risk for psychosis (CHR). The current study addressed this gap in the literature. We hypothesized that sleep disturbance would have a unique relationship with suicidal ideation/attempts when accounting for other variables in the model. We also hypothesized that the interaction between sleep disturbance/attenuated positive symptoms and sleep disturbance/stress would be related to suicidal ideation/attempts in CHR.

**Study Design:**

The current study used data generated by the Accelerating Medicines Partnership® Schizophrenia Observational Study. The total sample included 1,048 participants (827 CHR and 221 community controls). Participants completed measures of suicidal ideation/attempts, attenuated positive symptoms, depressive symptoms, perceived stress, and sleep disturbance.

**Study Results:**

Results supported a relationship between sleep disturbance and suicidal ideation/attempts in CHR, with participants who had lifetime ideation and attempts experiencing more sleep disturbance than those with no ideation or attempts. We also found small, but significant positive correlations between sleep disturbance and suicide risk in CHR. When accounting for other variables in the model, the effect of sleep disturbance remained significant for past month ideation, but not lifetime ideation or attempts. Both interaction models were non-significant.

**Conclusions:**

Our findings highlight the potential value of sleep measures in early identification and treatment of suicide risk in CHR. Further research in this area is warranted.

## Introduction

Individuals in the early course of psychotic disorders are at increased risk for suicide, with some evidence even suggesting that suicidality might serve as a transdiagnostic risk factor for the development of psychosis later in life.^1^ Consistent with this notion, individuals at clinical high risk for psychosis (CHR) are at increased risk for suicide, with over 50% experiencing recent suicidal ideation and approximately 25% making a lifetime suicide attempt.^2^^,^^3^ Despite the well-established finding that individuals at CHR are at increased risk for suicide, research identifying risk factors is still in its infancy.

Sleep disturbance (eg, restless sleep, difficulty falling asleep, poor sleep quality) is a well-established risk factor for suicide in the general population^4–6^ and among individuals with first-episode psychosis.^7^ Sleep disturbance is common across the psychosis continuum with individuals at CHR having similar or higher prevalence rates as individuals with both first-episode and longstanding psychosis.^8^^,^^9^ Given the high prevalence of sleep disturbance in CHR and the well-established link between sleep disturbance and suicide risk, researchers have also begun to examine the relationship between sleep and suicide risk in CHR. One study found that first-episode patients who experienced suicidal ideation during their prodromal phase also had higher rates of sleep disturbance during this same period of illness.^10^ Another study found that sleep deficiencies (ie, difficulty falling asleep, restless sleep) are related to suicidal ideation across the psychosis continuum, including among individuals at CHR.^9^ The largest study to date examined sleep disturbance and suicidal ideation in the NAPLS-3 CHR cohort,^11^ demonstrating a relationship among sleep quality, insomnia, sleep disturbance, and suicidal ideation. There was also a significant positive relationship between insomnia and suicide attempts.^11^ Taken together, these studies provide preliminary support for a relationship between sleep disturbance and suicide risk in CHR, warranting further investigation in additional large international samples.

In addition to suicide risk, sleep disturbance is associated with other important outcomes in CHR. For instance, studies have demonstrated a relationship between sleep disturbance and attenuated positive symptoms, worse functioning, and increased stress among individuals at CHR.^11–16^ Importantly, these same correlates of sleep disturbance in CHR are also related to suicide risk in this population.^10^^,^^17–22^ Thus, in addition to sleep disturbance potentially having a direct influence on suicide risk in CHR,^11^ it is also possible that sleep interacts with other variables to further exacerbate suicide risk. Two promising moderators for further study are severity of attenuated positive symptoms and stress. Specifically, studies have shown that attenuated positive symptoms might be related to both sleep disturbance^12^^,^^13^ and suicide risk in CHR.^17^^,^^22^ Similarly, studies have shown that stress intolerance is also positively associated with both sleep disturbance^14^^,^^16^ and suicide risk^20^^,^^21^ in CHR. However, no study to date has examined whether the interaction of sleep disturbance, attenuated positive symptoms, and/or stress is associated with suicide risk in this population.

Therefore, the purpose of the current study was to examine: (1) whether sleep disturbance differs among individuals at CHR with recent and/or lifetime suicide risk compared to those without, (2) whether sleep disturbance has a unique relationship with suicidal ideation and attempts when accounting for other variables that are relevant to sleep and/or suicide, and (3) the interaction between sleep disturbance/attenuated positive symptoms and sleep disturbance/perceived stress as it relates to suicide-risk in CHR. We hypothesized that (1) individuals at CHR with suicidal ideation and attempts would report greater recent sleep disturbance than those without, (2) sleep disturbance would be related to suicidal ideation and attempts when accounting for covariates, and (3) that the interaction between sleep disturbance/attenuated positive symptoms and sleep disturbance/stress would be related to suicidal ideation and attempts in CHR. More specifically, we hypothesized that (1) the relationship between positive symptoms and suicide outcomes would be strongest when sleep disturbance is more pronounced and (2) the relationship between stress and suicide outcomes would be strongest when sleep disturbance is more pronounced.

## Methods

### Participants and Procedures

The dataset used in this study was generated by the Accelerating Medicines Partnership® Schizophrenia (AMP® SCZ) Observational Study^23^ and was downloaded from the NIMH Data Archive (data release 2.0 DOI: 10.15154/8ax0-e673). AMP® SCZ is a multi-site, deep-phenotyping study with the primary goal of using multi-modal biomarkers (eg, EEG, MRI, genetics, language, cognition, etc.) to predict clinical trajectories of psychosis risk. Data collection involves 43 international partnering sites. The current study is a secondary data analysis of AMP® SCZ data. CHR status was determined using the *Positive Symptoms and Diagnostic Criteria for the CAARMS Harmonized with the SIPS*^24^ (PSYCHS). Full details about the study design, including primary/secondary hypotheses and inclusion/exclusion criteria for the CHR and community control groups, can be obtained elsewhere^23^^,^^25^ (https://www.ampscz.org/). The total sample included 1,048 participants (827 CHR and 221 community controls). Only participants with completed measures of interest were included in each analysis. The AMP® SCZ study was reviewed and approved by the Institutional Review Board at Northwell Health.

### Measures

#### Suicidal Ideation and Attempts

The *Columbia Suicide Severity Rating Scale*^26^ clinical interview was used to assess suicidal ideation and attempts. The first 2 items (ie, wish to be dead and non-specific active suicidal thoughts) were used to create binary variables to indicate the presence/absence of lifetime and past month suicidal ideation. Binary responses to the CSSRS “actual attempt” item determined the presence/absence of lifetime suicide attempt(s). These variables were also used to stratify individuals at CHR as no lifetime ideation/attempts, lifetime ideation only, and lifetime ideation plus attempts; participants were also stratified into past month ideation and no past month ideation.

#### Severity of Attenuated Positive Symptoms

The *Positive Symptoms and Diagnostic Criteria for the CAARMS Harmonized with the SIPS*^24^ interview assessed total severity of attenuated positive symptoms. The PSYCHS is a harmonized version of the 2 most widely used clinical interviews for assessing psychosis risk: Structured Interview for Psychosis-risk Syndromes^27^ and the Comprehensive Assessment of At-Risk Mental States.^28^ The current study used the total positive score (score 0-90) to measure overall attenuated positive symptom severity.

#### Depressive Symptoms

The *Calgary Depression Scale for Schizophrenia*^29^ clinical interview assessed past 2-week depressive symptoms. Items are rated on a 0-3 scale with higher scores indicating greater depressive symptom severity. For the current study, we calculated a total score excluding the suicide-risk item to prevent criterion contamination.

#### Perceived Stress

The *Perceived Stress Scale*^30^^,^^31^ assessed past month stress. The PSS is a 10-item self-report measure of the degree to which an individual perceives their circumstances as unpredictable, uncontrollable, and overwhelming. Items are rated on a 5-point Likert scale (*Never* to *Very Often*) with higher scores indicating a higher degree of perceived stress. Cronbach’s alpha for the entire sample was 0.88.

#### Sleep Disturbance

The self-report *PROMIS Sleep Disturbance* measure^32^^,^^33^ assessed past week sleep disturbance. The PROMIS-SD is an 8-item self-report measure of perceived sleep quality, satisfaction, and difficulty falling asleep. Items are rated on a 5-point Likert scale (*Not at all* to *Very Much*). Raw total scores are converted to T scores, with higher scores indicating more severe sleep disturbance. Cronbach’s alpha for the entire sample was 0.76.

### Statistical Analyses

Chi-square analyses were used to examine whether individuals at CHR have higher rates of suicidal ideation and attempts than community controls. Next, we used planned contrasts to compare sleep disturbance among CHR participants with no lifetime ideation/attempts, ideation only, and ideation plus attempts; we were unable to include an attempt only group because only one CHR participant endorsed a lifetime attempt without lifetime ideation. Similarly, we compared sleep disturbance between CHR participants with and without past month ideation using a *t-*test; we were unable to stratify into 3 groups (none, ideation only, ideation plus attempts) because (1) recent ideation and attempts were assessed on different timescales (past month ideation and past 3 month attempts) and (2) there were too few recent attempts to include in our analyses.

Next, we examined correlations between all variables (suicidal ideation, suicide attempts, depression, attenuated positive symptoms, stress, sleep disturbance) in the CHR group only using Pearson correlations (r_pb_ for continuous-binary variables). Logistic regression was used to examine whether sleep disturbance is significantly related to suicidal ideation and attempts when accounting for other variables in the model. Logistic regression models also examined the interaction between sleep/attenuated positive symptoms and sleep/stress. All assumptions were tested prior to analysis. Depression violated the linearity of logit assumption for all past month ideation models. Therefore, we also conducted sensitivity analyses using general additive models for past month ideation. Results were largely the same and are available upon request. Additionally, demographic variables were examined as potential covariates for inclusion in our models. Using a-priori criteria (*P* < .10), we determined that race and sex would be included as covariates. All logistic regression models were conducted with the CHR subsample only, as our overarching aim was to examine the relationship between sleep disturbance and suicide risk in CHR. Missing data was handled using listwise deletion; Little’s MCAR test confirmed that data were missing at random. Effect sizes are reported using Cohen’s d (planned contrasts), Cramer’s V (chi-square), and odds ratios (logistic regression).

## Results

**Figure 1:**
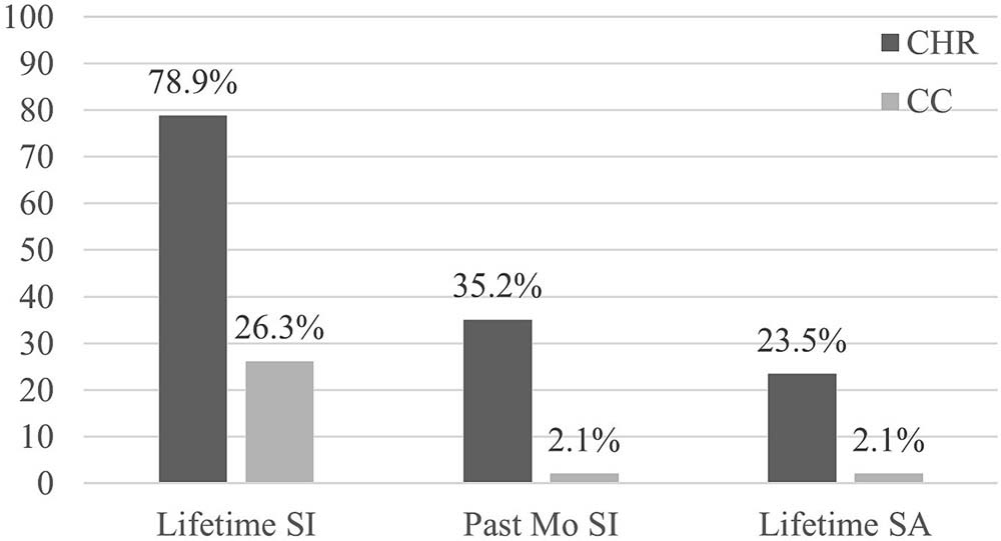
Rates of Suicidal Ideation and Attempts in CHR Compared to Community Controls. Note: SI = Suicidal Ideation, SA = Suicide Attempt, CHR = Clinical High Risk for Psychosis, CC = Community control. 892 participants had lifetime ideation data (CHR = 698, CC = 194). 885 participants had past month ideation data (CHR = 694; CC = 191). 880 participants had lifetime attempt data (CHR = 689; CC = 191)

Demographic characteristics are presented in Table 1. The sample was predominantly white, female, and non-Hispanic. Results from the Chi-square analyses indicate that the CHR group had higher rates of lifetime ideation (χ^2^(1) = 191.80, *P* < .001, V = 0.46), past month ideation (χ^2^(1) = 81.18, *P* < .001, V = 0.30), and lifetime suicide attempts (χ^2^(1) = 44.82, *P* < .001, V = 0.23) compared to community controls. Specifically, 78.9% of the CHR group had lifetime ideation, 35.2% had past month ideation, and 23.5% had lifetime attempts (Figure 1).

**Table 1:** Participant Characteristics

	**Total (*n* = 1048)**		**CHR (*n* = 827)**		**CC (*n* = 221)**	
**Age (*M* ± SD)**	20.67 ± 4.06		20.40 ± 4.09		21.68 ± 3.80	
**Sex assigned at birth**						
Male	402	38.4%	308	37.2%	94	42.5%
Female	646	61.6%	519	62.8%	127	57.5%
**Race**						
White	616	58.8%	488	59.0%	128	57.9%
Black or African American	92	8.8%	84	10.2%	8	3.6%
Asian	197	18.8%	134	16.2%	63	28.5%
American Indian/Alaskan Native	15	1.4%	11	1.3%	4	1.8%
Hawaiian or Pacific Islander	2	0.2%	2	0.2%	12	5.4%
More than one race	86	8.2%	74	8.9%	6	2.7%
Unknown or not reported	40	3.8%	34	4.1%	128	57.9%
**Ethnicity**						
Non-Hispanic	823	78.5%	645	78.0%	178	80.5%
Hispanic	130	12.4%	106	12.8%	24	10.9%
Unknown or not reported	95	9.1%	76	9.2%	19	8.6%

Abbreviations: CC = community control; CHR = clinical high risk for psychosis.

### Relationship between Sleep Disturbance and Suicidal Ideation/Attempts in CHR

Planned contrasts revealed that participants with lifetime ideation and attempts had more sleep disturbance than those with no lifetime ideation or attempts (*t*(660) = 4.43, *P* < .001, *d* = 0.52). They also had more sleep disturbance than those with ideation only (*t*(660) = 3.06,  *P* = .002, *d* = 0.29). Individuals at CHR with lifetime ideation only had more sleep disturbance than those with no lifetime ideation or attempts (*t*(660) = 2.27, *P* = .023, *d* = 0.23). CHR participants with past month ideation had greater sleep disturbance than those without (*t*(665) = 5.63, *P* < .001, *d* = 0.45).

Correlations are summarized in Table 2. Results indicate a small, but significant relationship between sleep disturbance and suicide risk. There were also small, but significant correlations between positive symptoms, stress, and suicide risk. Logistic regression models examining the relationship between sleep and suicidal ideation/attempts when accounting for relevant variables in the model (demographics, depression, attenuated positive symptoms, and stress) are summarized in Table 3. For the lifetime ideation model, only race and depression were significant. The relationship between sleep disturbance and lifetime ideation was not significant (OR = 1.034, 95% CI, 0.994-1.075). For the lifetime attempt model, race, depression, and attenuated positive symptoms were significant. The relationship between sleep disturbance and lifetime attempts was not significant (OR = 1.037, 95% CI, 0.999-1.077). In contrast, the relationship between sleep disturbance and past month ideation was significant even with other relevant variables in the model (OR = 1.044, 95% CI, 1.007-1.084); depression and attenuated positive symptoms were also significant in this model.

**Table 2:** Correlations in the CHR Group

	**1**	**2**	**3**	**4**	**5**	**6**
1. Lifetime SI	–					
2. Past Month SI	0.38^**^	–				
3. Lifetime SA	0.28^**^	0.25^**^	–			
4. Depression	0.29^**^	0.46^**^	0.20^**^	–		
5. Positive Sx	0.17^**^	0.23^**^	0.24^**^	0.27^**^	–	
6. Stress	0.24^**^	0.33^**^	0.16^**^	0.53^**^	0.29^**^	–
7. Sleep Dist	0.14^**^	0.21^**^	0.15^**^	0.22^**^	0.16^**^	0.31^**^

Abbreviations: Positive Sx = Attenuated Positive Symptoms; SA = Suicide Attempts; SI = Suicidal Ideation; Sleep Dist = Sleep Disturbance. ^**^Correlation is significant at the 0.01 level (2-tailed).

**Table 3:** Is Sleep Disturbance Related to Suicide Outcomes in CHR when Accounting for Other Variables?

	Lifetime ideation (*n* = 581)			Past month ideation (*n* = 578)			Lifetime attempts (*n* = 576)		
	B	*P*	OR	B	*P*	OR	B	*P*	OR
Sex	0.250	.264	1.284	−0.072	.731	0.931	0.377	.095	1.458
Race	−0.483	.027	0.617	−0.255	.218	0.775	−0.545	.016	0.580
Depression	0.199	<.001	1.220	0.219	<.001	1.244	0.086	.003	1.090
Positive Sx	0.026	.054	1.026	0.023	.039	1.023	0.056	<.001	1.058
Stress	0.006	.758	1.006	0.024	.189	1.024	−0.020	.291	0.980
Sleep Dist	0.033	.100	1.034	0.043	.021	1.044	0.037	.058	1.037

Abbreviations: Positive Sx = attenuated positive symptoms; Sleep Dist = sleep disturbance.

### Interaction between Sleep, Attenuated Positive Symptoms, and Stress in CHR

Table 4 summarizes models examining the interaction between sleep disturbance and attenuated positive symptoms. Results were non-significant for lifetime ideation (OR = 0.998, 95% CI, 0.993-1.002), past month ideation (OR = 1.001, 95% CI, 0.997-1.005), and lifetime attempts (OR = 1.002, 95% CI, 0.998-1.007). Table 4 also summarizes models examining the interaction between sleep and stress. Results were non-significant for lifetime ideation (OR = 1.004, 95% CI, 0.998-1.010), past month ideation (OR = 1.000, 95% CI, 0.994-1.005), and lifetime attempts (OR = 0.999, 95% CI, 0.994-1.004).

**Table 4:** Does Sleep Disturbance Interact with Attenuated Positive Symptoms or Perceived Stress?

	Lifetime ideation (*n* = 581)			Past month ideation (*n* = 578)			Lifetime attempts (*n* = 576)		
	B	*P*	OR	B	*P*	OR	B	*P*	OR
**Model 1**									
Sex	0.264	.239	1.302	−0.079	.707	0.924	0.376	.097	1.457
Race	−0.503	.022	0.605	−0.254	.221	0.776	−0.546	.016	0.579
Depression	0.199	<.001	1.220	0.219	<.001	1.245	0.086	.003	1.090
Stress	0.006	.740	1.006	0.024	.192	1.024	−0.021	.279	0.979
Positive Sx	0.157	.202	1.170	−0.029	.803	0.972	−0.079	.494	0.924
Sleep	0.079	.099	1.083	0.022	.658	1.023	−0.023	.666	0.977
Sleep × Positive Sx	−0.002	.281	0.998	0.001	.655	1.001	0.002	.240	1.002
**Model 2**									
Sex	0.257	.252	1.292	−0.073	.726	0.929	0.372	.100	1.451
Race	−0.468	.033	0.626	−0.256	.217	0.774	−0.545	.016	0.580
Depression	0.199	<.001	1.220	0.219	<.001	1.244	0.086	.003	1.090
Positive Sx	0.025	.058	1.026	0.023	.038	1.023	0.057	<.001	1.058
Stress	−0.208	.210	0.812	0.044	.773	1.045	0.041	.781	1.042
Sleep	−0.045	.473	0.956	0.052	.444	1.054	0.063	.340	1.065
Sleep × Stress	0.004	.195	1.004	0.000	.894	1.000	−0.001	.674	0.999

Abbreviations: Positive Sx = attenuated positive symptoms; Sleep × Positive Sx = interaction between sleep and attenuated positive symptoms; Stress × Sleep = interaction between sleep and stress.

## Discussion

Consistent with prior research,^2^^,^^3^ the current study demonstrates that individuals at CHR are high risk for suicide, with nearly 80% of our sample experiencing lifetime suicidal ideation and nearly 25% attempting suicide in their lifetime. Rates of recent ideation were also high, with over one-third of CHR participants endorsing suicidal thoughts within the past month. These prevalence estimates were significantly higher than community controls, with moderate effect sizes for all group comparisons. Thus, there is a critical need for research that identifies factors that contribute to suicidal thoughts and behaviors in this high-risk population.

The current study examined a promising yet understudied risk factor for suicide in CHR—sleep disturbance. Results generally supported a relationship between sleep disturbance and suicidal ideation/attempts in CHR, with participants who had lifetime ideation and attempts experiencing more sleep disturbance than those with no ideation or attempts. Similarly, we found small, but significant positive correlations between sleep disturbance and all three suicide outcomes in CHR. When accounting for other relevant variables in the model, the effect of sleep disturbance remained significant for past month suicidal ideation, but not lifetime ideation or attempts. These findings add to the growing body of literature supporting a relationship between sleep disturbance and suicide risk in CHR.^9–11^ Specifically, Andriopoulos and colleagues found that patients with suicidal ideation during their prodromal phase also reported more sleep difficulties during that same phase of illness. Similarly, Cohen and colleagues found that sleep disturbance, sleep quality, and insomnia are related to suicidal ideation in CHR; insomnia was also related to suicide attempts. Our prior work has also demonstrated a relationship between sleep deficiencies and suicidal ideation across the psychosis continuum, including individuals at CHR.^9^ Of note, the one study to date that did not find a significant relationship between sleep and suicide risk used a measure that combined lifetime attempts, lifetime ideation, and recent ideation into one total suicide risk score.^18^ The current study might explain this discrepant finding, as we found that sleep only remained significant in the adjusted models for past month suicidal ideation, not lifetime ideation/attempts, suggesting that sleep disturbance might have a stronger relationship with recent ideation (ie, past month) than lifetime ideation/attempts. Critically, sleep disturbances can fluctuate substantially over time^34^ and the time discrepancy between lifetime suicide outcomes and recent sleep disturbance precludes the ability to make inferences about sleep at the time of the attempt. Thus, measures that assess similar, proximal timeframes (ie, recent ideation and recent sleep disturbance) may be better suited to answer questions about the relationship between sleep disturbance and suicide risk in CHR, whereas measures that assess discrepant, distal timeframes (ie, lifetime ideation/attempt and recent sleep disturbance) answer questions about whether individuals at CHR with a history of suicide risk are more likely to have recent sleep disturbance than those without. It is possible that sleep disturbance is a proximal risk factor for suicide in CHR and studies that utilize measures with similar, proximal timeframes will find stronger effects than those with distal and/or discrepant timeframes. Future studies that use intensive longitudinal designs, such as actigraphy paired with ecological momentary assessment, would be ideally suited to examine whether sleep has a direct, proximal impact on suicide risk in CHR; such work is currently underway through other AMP® SCZ projects.

Contrary to our hypotheses, we did not find a significant interaction effect between sleep/stress or sleep/attenuated positive symptoms on suicidal ideation or attempts in CHR. Thus, it is possible that sleep independently contributes to suicide risk in CHR. Importantly, however, because of our cross-sectional design, we only examined sleep as a moderator (ie, whether the relationship between attenuated symptoms/stress and suicide risk is most pronounced when sleep is poor). It remains possible that sleep contributes to the relationship between symptoms, stress, and suicide risk in other ways. For instance, studies have shown that sleep directly affects positive symptoms^14^^,^^35^^,^^36^ and stress.^14^^,^^37^ Thus, it is possible that poor sleep leads to a heightened stress response and more positive symptoms, contributing to suicide risk through an indirect pathway (ie, positive symptoms and stress might mediate the relationship between sleep and suicide outcomes). Alternatively, it is also possible that these are third variables that lead to changes in both sleep and suicide risk in CHR. Future longitudinal studies are needed to examine possible mediation models of sleep, symptoms, stress, and suicide risk in CHR.

Although the primary aim of the current study was to examine the relationship between sleep disturbance and suicidal ideation/attempts in CHR, several other noteworthy findings emerged. Specifically, correlations supported a relationship between suicidal ideation/attempts and depressive symptoms, attenuated positive symptoms, and stress. In the full models, depression remained significant, but stress did not. Attenuated positive symptoms remained significant for past month ideation and lifetime attempts, but not lifetime ideation. Our somewhat mixed findings regarding the relationship between attenuated positive symptoms and suicide outcomes in CHR aligns with the broader inconsistent literature in this area.^17–22^^,^^38^^,^^39^ As suggested in prior studies, it is possible that the relationship between attenuated positive symptoms and suicide in CHR is explained by third variables^22^^,^^40^ and/or that this relationship exists only for certain people (ie, there are unidentified moderators influencing the relationship).^41^ Further research is needed to test these possibilities.

A major strength of the current study is the large, international sample. Nonetheless, the following limitations should be considered when interpreting our results. First, the current study used a cross-sectional design, which prevents us from making inferences about the prospective and/or causal nature of the relationship between sleep disturbance and suicide risk in CHR; future longitudinal studies are needed for detailed mechanistic investigations in this area. Our cross-sectional design also precludes our ability to make inferences about the direction of this relationship, as it is also plausible that suicidal ideation, which often occurs at night,^42^ leads to greater sleep disturbance. Second, the current study assessed past week sleep disturbance, past month ideation, and lifetime ideation/attempts, resulting in a temporal mismatch between our variables of interest. This temporal mismatch limits the inferences we can make from this study, as we were unable to examine whether sleep is a short-term or proximal risk factor for suicidal ideation and/or attempts. Future ambulatory studies are needed to examine whether sleep directly influences suicide risk during the night of poor sleep and/or during the subsequent day. Third, the current study assessed sleep disturbance using a self-report measure, which is limited by individual differences in insight and perceptions of sleep. Additional research using objective sleep measures (eg, actigraphy, polysomnography) are needed to validate our findings. Finally, sleep is a multi-faceted construct and the current study was limited by our broad focus on sleep disturbance. Additional research is needed to determine which specific aspects of sleep disturbance (eg, poor sleep efficiency, insomnia, hypersomnia, etc.) are related to suicide outcomes among individuals at CHR. Notwithstanding these limitations, this study supports a relationship between sleep disturbance and suicide risk in CHR. With further validation and additional longitudinal research, our findings highlight the potential value of sleep measures in early identification of suicide risk in CHR. Additionally, sleep might be a useful therapeutic target for reducing suicide risk in this population. Further research in this area is warranted.

## Data Availability

The data that support the findings of this study are publicly available through the NIMH Data Archive.
